# Structural damage detection using deep learning and FE model updating techniques

**DOI:** 10.1038/s41598-023-46141-9

**Published:** 2023-10-31

**Authors:** Yunwoo Lee, Heesoo Kim, Seongi Min, Hyungchul Yoon

**Affiliations:** 1 School of Civil Engineering, College of Engineering, Chungbuk National University, Cheongju, Republic of Korea; 2 Future and Fusion Lab of Architectural, Civil and Environmental Engineering, Korea University, Seoul, Republic of Korea; 3 School of Civil, Environmental, and Architectural Engineering, Korea University, Seoul, Republic of Korea

**Keywords:** Civil engineering, Computational science

## Abstract

The structural condition can be estimated by various methods. Damage detection, as one of those methods, deals with identifying changes in specific features within structural behavior based on numerical models. Since the method is based on simulation for various damage conditions, there are limitations in applicability due to inevitable discrepancies between the analytical model and the actual structure. Finite element model updating is a technique for establishing a finite element model that can reflect the current state of a target structure based on the measured responses. It is performed based on optimization for various structural parameters, but the final output can converge differently depending on the initial model and the characteristics of the algorithm. Although the updated model may not faithfully replicate the target structure as it is, it can be considered equivalent in terms of the relationship between the structural properties and behavioral characteristics of the target. This allows for the analysis of changes in the mechanical relationships established for the target structure. The change can be related to structural damage, and artificial intelligence technology can provide an alternative solution in such complex problems where analytical approaches are challenging. Taking practical aspects from the aforementioned methods, a novel structural damage detection methodology is presented in this study for identifying the location and extent of the damage. Model updating is used to establish a reference model that reflects the structural characteristics of the target. Training data for various damage conditions based on the reference model allows the artificial intelligence networks to identify damage to the target structure.

## Introduction

There are various methods for assessing the as-is condition of structures. Most popular methods that are being utilized in the sites are on-site manual inspections and vibration-based structural health monitoring. On-site inspections involve visual inspection and manual assessment of structural elements to detect visible damages or anomalies^1–3^. While it provides a useful means of assessing the condition of structure, the accuracy and reliability of the assessment can be subjective and prone to human errors. Additionally, on-site inspections are typically conducted at specific intervals, which means that the assessment may not capture the real-time behavior or deterioration of the structure continuously. Vibration-based structural health monitoring systems, on the other hand, can continuously monitor the behavior and response of structures using sensors and data acquisition techniques^4–8^. Structural health monitoring systems analyzes the collected data and performs post processing to obtain useful information that could refer to the condition of the structure^9–13^. These methods offer a more comprehensive and objective assessment of the structural condition, can provide preventive maintenance and timely responses to maintain structural integrity and safety.

The structural response can be analyzed using various methods to evaluate their condition. One of the most commonly employed approaches is vibration-based structural system identification, which aims to extract modal parameters and dynamic characteristics of structures through the analysis of measured vibrations. By measuring the dynamic characteristics, valuable insights into the structural integrity can be obtained. The advantages of this method include its non-destructive nature, simplicity of application, and the ability to capture global responses. There are a number of studies that attempted to estimate the change of structural conditions through changes in natural frequencies using long-term measurement data^14–18^. While some studies have shown that the natural frequency can provide valuable information related to the stiffness of structures, it was not sufficient to estimate the condition of structures directly. For most studies, the natural frequency was rather utilized as the input parameter (feature) of a system that can be used to evaluate the structural integrity indirectly.

Another method that are being widely studied for vibration-based structural health monitoring is damage detection. Damage detection methods not only finds the location of the damages, but also estimates the magnitude of the damage. Most of the methods utilize the differences in structural responses under damaged and intact conditions to detect the damage. Various responses can be used to identify the behavioral characteristics of the structure including acceleration^19–21^, strain^22–24^, displacement^25–27^. Once the response of the structure is measured, advanced techniques including signal processing algorithms^28–30^, and machine learning^31–33^, can be utilized to detect the damage. While dynamic characteristics such as natural frequency and mode shape are generally being used as input parameters for these post processing procedure, many researchers have used natural frequencies as a basis for identification of structural integrity^34–36^. In conventional methods, a specific damage index was proposed so that the characteristics of the change in natural frequency for the damaged condition can be emphasized^37–39^. However, these methods were difficult to derive an index that can sufficiently represent the magnitude and location of damage from the natural frequency through a kind of numerical calculation process. Moreover, since the method is performed based on simulation, there were limitations to its application due to discrepancies in the behavior of actual structures.

Finite element (FE) model updating is an additional way to assess the current state of structures^40–44^. This method utilizes FE models to refine and update the numerical model, aiming to achieve a more accurate representation of the systematic behavior of the target structure. Parameters for the model can include material properties, boundary conditions, or geometric features. This process typically employs an optimization algorithm that minimizes the discrepancy between the simulated responses of the model and the measured responses from sensors or experimental tests. By iteratively adjusting the model, a closer alignment with the behavior of the target structure is achieved. The natural frequency of the structure serves as a prominent input feature within this method as well. Since the natural frequency is one of the most effective factors that can represent the relationship between the stiffness and mass of a structure, it can allow the FE model to converge towards the mechanical characteristics of the target structure. However, the updated model finally converged depends on the initial model and the characteristics of the optimization algorithm, as well as the assumptions within the updating process. In addition, there is a limitation for constructing a model that can replicate all the behavioral characteristics of the structure, including static behavior, solely based on dynamic characteristics. Nevertheless, it can still present a possibility of being used to identify changes in such established relationships by optimizing the relationship between the considered structural properties and behavioral characteristics.

In an effort to address the challenges in a conventional method of structural damage detection, researchers have investigated the application of artificial intelligence (AI) technology. AI technology can provide alternative data-driven solutions for problems that involve hard-to-detect patterns or those that cannot be deterministically defined^45^. The efficiency of the solutions is closely reliant on the quality of the training data. As technology for generating and processing massive data has been developed, its usability has been significantly enhanced and it is being applied to various fields. In terms of the architectural aspect of the network, it has been utilized in various forms for structural damage detection. Deep neural network (DNN)^46–48^, as the fundamental form of a neural network, explores the relationship between input and output through a feed-forward process. It is useful when input and output can be relatively simply defined. Recurrent neural network (RNN)^49–51^, with its recurrent structure capable of incorporating past information into the current state, is suitable for processing sequential data such as time series data. Data such as the dynamic response of structures can be used as the input for RNN. Convolutional neural network (CNN)^52–54^ is primarily specialized in processing spatial data such as images. It is used for extracting spatial features from data, and is not only useful for image analysis but also facilitates the extension of data features for the analysis. In terms of training methodology, there are supervised methods where outputs are labeled for corresponding inputs to extract features for the relationship, and unsupervised methods^55–57^ that discover patterns from data without labels and automatically train networks. Supervised methods, being based on clear labels, can provide excellent performance for accurate predictions. However, in cases where it is challenging to specify clear labels, such as in the conditions of real structures, or when the goal is to discover new features, unsupervised methods may be more appropriate.

This study adopts the practical aspects of the previously described techniques for efficient application to damage detection in actual structures. An FE model updating technique was applied to construct an FE model capable of appropriately representing the dynamic characteristics of the target structure. Subsequently, structural analysis simulations were conducted for various arbitrary damage cases based on the updated FE model. Artificial intelligence technology can be trained on various damage conditions that can occur in the target structure through simulated data. Given that the reference FE model was designed to reflect the target structure faithfully, the trained network can effectively identify the location and degree of damage in the target structure when it occurs.

The following chapter provides a description of the proposed methodology. The feasibility of the proposed method has been verified through experimental tests, and the relevant comprehensive discussion is given in section “Validation test”, including structural analysis simulations and the training of artificial intelligence network. Section “Conclusion” covers a brief conclusion and future work for the study.

## Methodology

**Figure 1:**
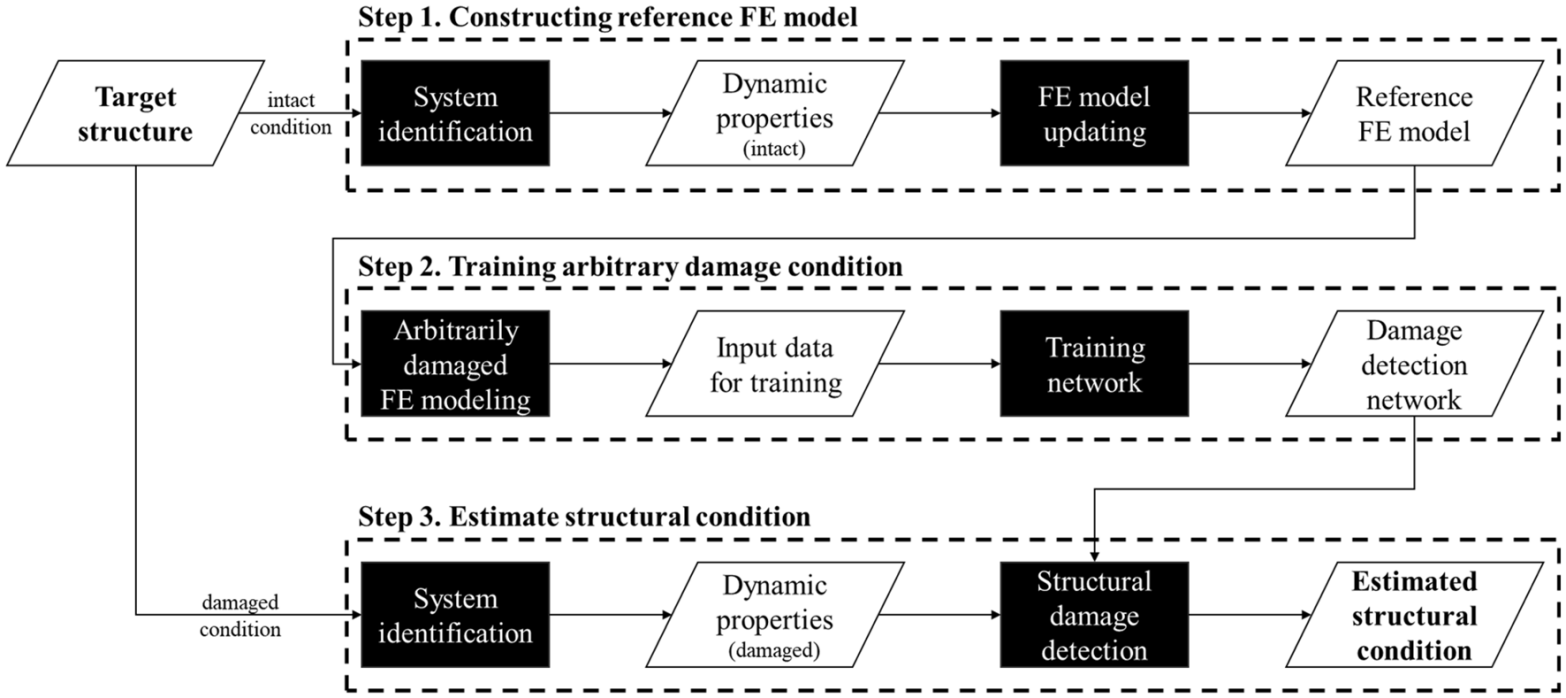
Schematic description of proposed method.

A schematic description of the proposed method in this study is shown in Fig. 1. The proposed method can be divided into three steps. The first step is to construct a reference FE model that can adequately represent the current state of the target structure. This can be achieved through FE model updating using dynamic properties obtained from system identification for the target structure. In the second step, numerous arbitrarily damaged FE models are generated based on the reference FE model derived in the previous step. These models are used to train the damage detection network for various damage conditions. System identification in the final stage, when defects occur in the structure, captures the dynamic characteristics of the damaged structure. Subsequently, this information is applied to the damage detection network to assess the current condition of the structure. Further details of the methodology are described through following sections.

### System identification

**Figure 2:**
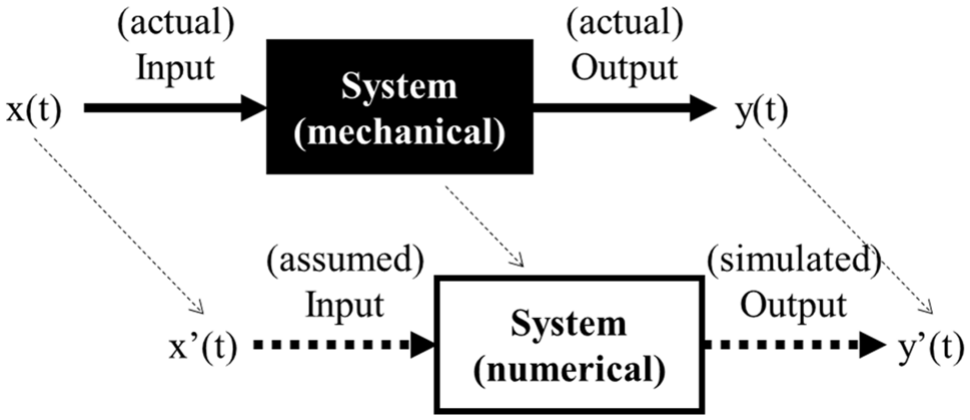
Basic concept of system identification.

System identification refers to the process of determining a mathematical model or characteristics of a system based on observed input and output data, as shown in Fig. 2. A system determines the relationship between input and output. When it is difficult to know the system through analytical approaches, the relationships between input and output data can be used to identify characteristics of the system and establish a corresponding mathematical system model. However, in case where the input to the system is not clear, such as in typical structures with complexly acting loads, output-only system identification can be conducted by assuming the input as white noise and using only the response of the structure.

System identification can be performed in several ways. In structural engineering, it is generally performed through frequency domain analysis of the ambient vibration response of the structure. The dynamic response data of the structure is transformed using techniques such as Fourier transform to derive frequency components, and then applying the peak-picking method, dominant peaks or resonant frequencies can be identified. For the processing of typical digital signals, the discrete Fourier transform as expressed in Eq. (1) is applied, allowing for the extraction of frequency components from the dynamic response data of structures.

1 $$ X(k) = \sum\limits_{n = 0}^{N - 1} {x(n)e^{ - i2\pi kn/N} } ,\quad (0 \le k < N) $$

Here, *x* is the input data to be transformed to the frequency domain, *N* is the number of samples of the input, and *k* denotes the frequency index and has values from 0 to *N* − 1.

Frequency Response Function (FRF) is one of the fundamental concepts in the field of structural dynamics, which represents the relationship between the input excitation and the corresponding output response in the frequency domain. It provides significant information for the dynamic behavior of a system and is widely used in various engineering applications. Defining $X(\omega )$ and $Y(\omega )$ as the Fourier transforms of the input and output signals, respectively, the FRF of the system can be expressed as Eq. (2).

2 $$H(\omega )=Y(\omega )/X(\omega )$$

The FRF serves as a valuable tool for system identification, allowing that the system's resonant frequencies, damping properties, and frequency-dependent behaviors can be discerned. This study used the FRF to identify the dynamic characteristics and extract the natural frequencies of the target structure.

### FE model updating

The FE model is a numerical representation of a physical structure used in engineering analysis and simulations. It divides the structure into smaller elements, enabling the complex structural behavior to be analyzed through smaller and more easily comprehensible elements. However, the FE model cannot reflect the characteristics of the target structure as it is, since there are inevitable differences between the actual structure and the theoretical FE model in terms of geometrical properties, material properties, and boundary conditions. In this regard, an FE model updating that minimizes the error between the target structure and the FE model is required.

**Figure 3:**
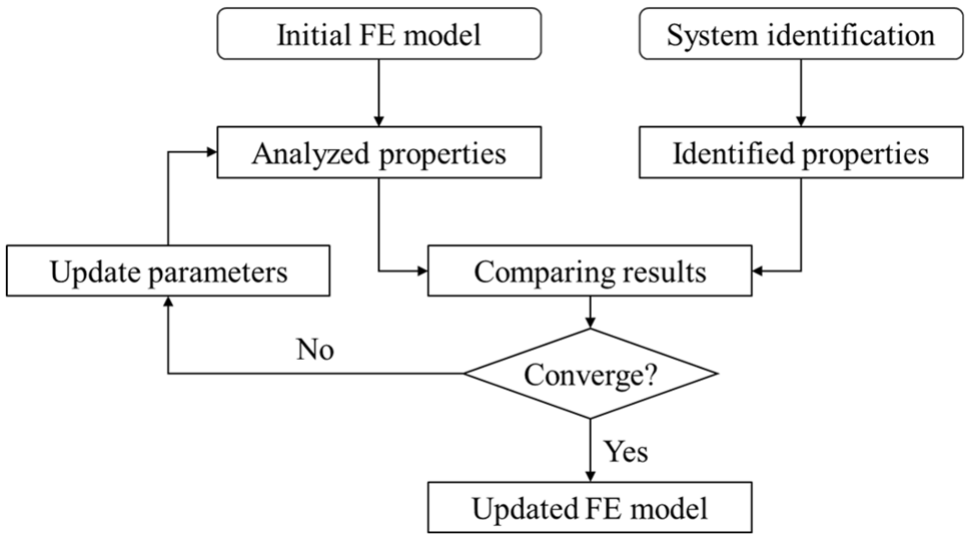
Basic procedure for FE model updating.

FE model updating is a process used to improve the accuracy of numerical FE models by adjusting their parameters based on experimental data, as shown in Fig. 3. In FE model updating, the initial FE model is compared with measured data obtained from sensors installed on the structure. By iteratively adjusting the model parameters, such as material properties, boundary conditions, or geometric properties, the updated FE model can better represent the dynamic behavior of the real structure. Model updating techniques generally involve optimization algorithms that minimize the objective function, which quantifies the differences between the FE model and the experimental measurements. The objective function is mainly set using the dynamic characteristics of the structure, such as the natural frequency. Although the FE model updated using only the natural frequencies cannot represent all the behaviors of the target structure as it is, it can be used to identify the changes in dynamic characteristics due to changes in the structural system. Once an updated FE model that can faithfully represent the dynamic characteristics of the target structure in its current state is established, the dynamic characteristics of subsequent structural defects can also be expressed based on the reference FE model. This study adopts the model updating technique to train a damage detection network by generating simulation data for various potential defects based on the reference FE model that replicates the dynamic characteristics of the target structural system.

### Deep neural network

The main objective of this study is to identify damage in structures through changes in natural frequencies, which are one of the dynamic characteristics of structures. The natural frequency is a parameter which directly related to the stiffness and mass of a structure. It provides meaningful information to identify damage that can be represented as changes in stiffness. However, the natural frequency represents the global characteristics of the structure, which depends on complex interactions of various factors within the structure, and is determined differently for each structural system. Particularly for complex structures, analyzing the relationship between these factors becomes more challenging, making it difficult to evaluate changes in stiffness through the natural frequency based on an analytical approach. This study used a deep neural network (DNN), an artificial intelligence network capable of providing alternative solutions based on data, to overcome the limitation of such analytical approaches.

**Figure 4:**
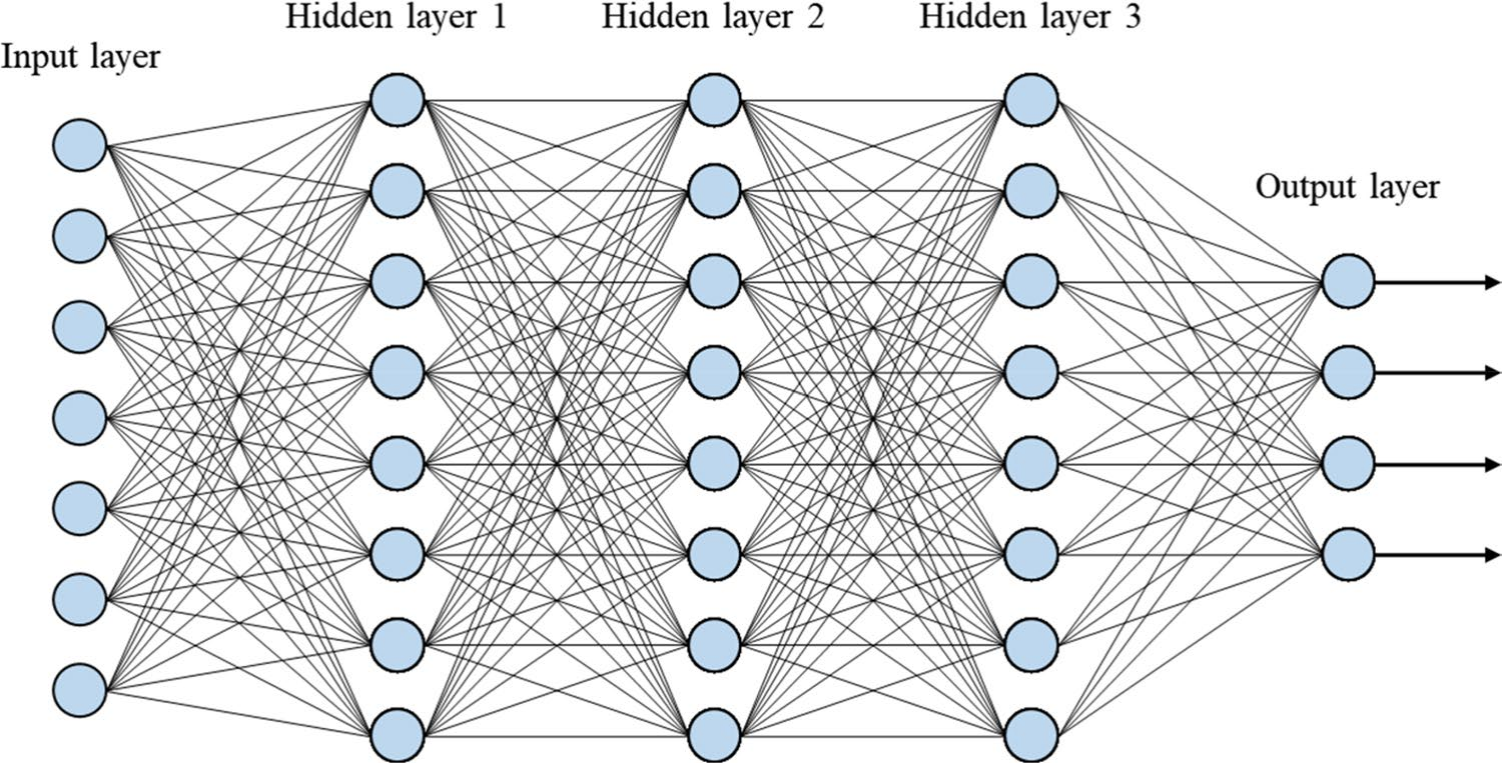
Deep neural network architecture.

As shown in Fig. 4, a DNN is composed of multiple layers, including an input layer, one or more hidden layers, and an output layer. The input layer receives the data or features for training, which are then processed through the hidden layers. The hidden layers contain interconnected nodes for extracting complex patterns. A node receives inputs, applies a weighted sum operation, and then derives a feature value at the node through a nonlinear activation function. This can be represented mathematically as Eq. (3).

3 $$\begin{array}{l}z=\sum w*x+b\\ h=\sigma (z)\end{array}$$

In the above, *z* represents the weighted sum of inputs (*x*) and their corresponding weights (*w*), *b* is the bias term, $\sigma$ denotes the activation function, and *h* represents the output of the node.

By stacking multiple layers, DNNs can learn hierarchical representations of data, allowing for the extraction of complex features. The information flow through the network can be expressed as Eq. (4)

4 $$ \begin{array}{*{20}c}   h_{1}  = \sigma \left( {w_{1} *x + b_{1} } \right) \\   h_{2}  = \sigma \left( {w_{2} *h_{1}  + b_{2} } \right) \\    \vdots  \\   h_{n}  = \sigma \left( {w_{n} *h_{{n - 1}}  + b_{n} } \right) \\  \end{array}  $$

Here, ${h}_{i}$ represents the output of layer $i$, ${w}_{i}$ denotes the weight matrix connecting layer $i-1$ to layer $i$, and ${b}_{i}$ is the bias vector of layer $i$. Finally, the output layer produces the desired output as Eq. (5) based on the processed information from the previous layers.

5 $$y=\sigma \left({w}_{n+1}*{h}_{n}+{b}_{n+1}\right)$$

Training a DNN involves optimizing its parameters (weights and biases) to minimize the discrepancy between predicted outputs and ground truth labels. This is achieved through a process called backpropagation, which computes the gradient of a defined loss function with respect to the parameters of the network. By applying an optimization algorithm, such as stochastic gradient descent, the network iteratively updates its parameters to improve its prediction accuracy.

## Validation test

The feasibility of the proposed method was evaluated based on the experimental test. Vibrational tests were conducted to extract the dynamic characteristics of the target structure, which were then used to establish a reference FE model that adequately represents the dynamic characteristics of the target structure. Based on the reference FE model, various FE models for arbitrary damage conditions were generated and used for training the deep learning network. The performance of the trained network was evaluated by applying the dynamic characteristics extracted through the test on intentionally damaged structures to the network.

### Experimental test

#### Experimental setup

**Figure 5:**
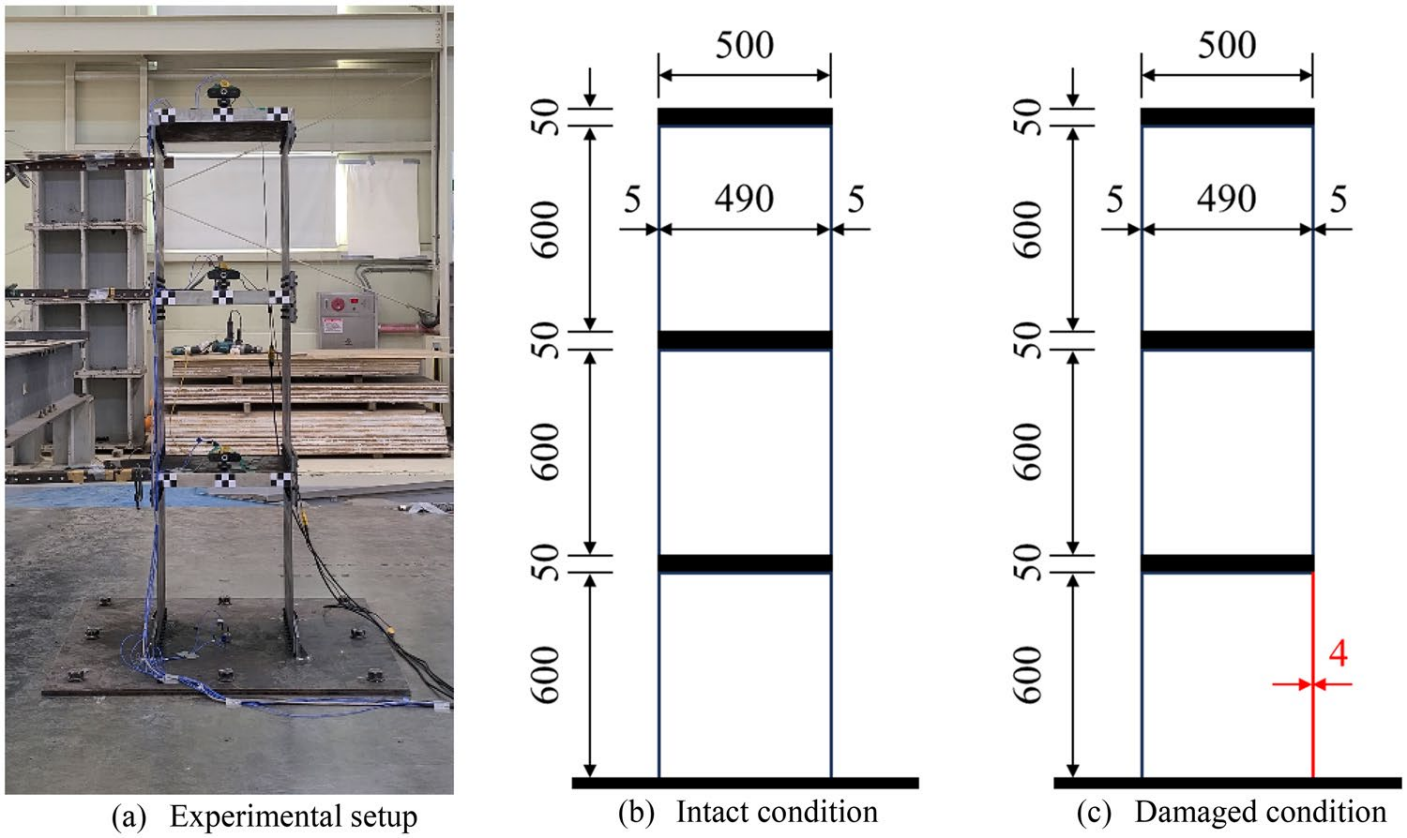
Target structure in validation test.

The experimental test was conducted on a three-story frame structure as shown in Fig. 5. The width and depth of the structure are both 500 mm, and the total height is 1950 mm. Each floor consists of a steel plate with a thickness of 50 mm, which is supported by wall plates with a height of 600 mm and a thickness of 5 mm. Structural damaged condition was considered by reducing the thickness on one side of the first-floor wall plate by 20%.

**Figure 6:**
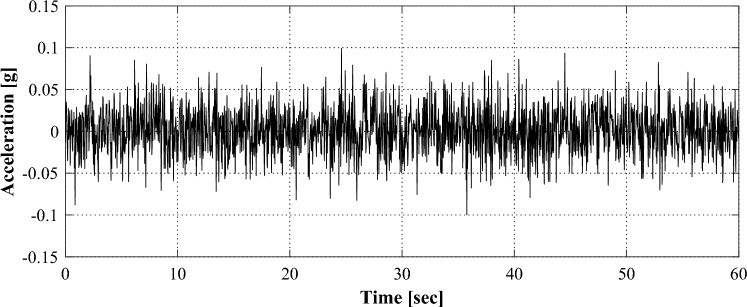
Excited base motion.

The dynamic behavior of the structure was induced by applying ground motion using a shaking table, so that the dynamic characteristics of the structure can be extracted. When ground motion has characteristics in a specific frequency range, it can disturb identifying the dynamic characteristics of the structure. Therefore, the applied ground motion was designed to have characteristics of white noise as shown in Fig. 6. The excitation lasted for 60 s, and the peak ground acceleration was applied as about 0.1 g.

The structural responses were measured by applying Yoon's method^50^ of vision-based displacement measurement, using a camera installed in front of the structure. The lateral displacement of the steel plate of each floor was extracted using a camera device with a frame rate of 30 fps.

#### Experiment results

**Figure 7:**
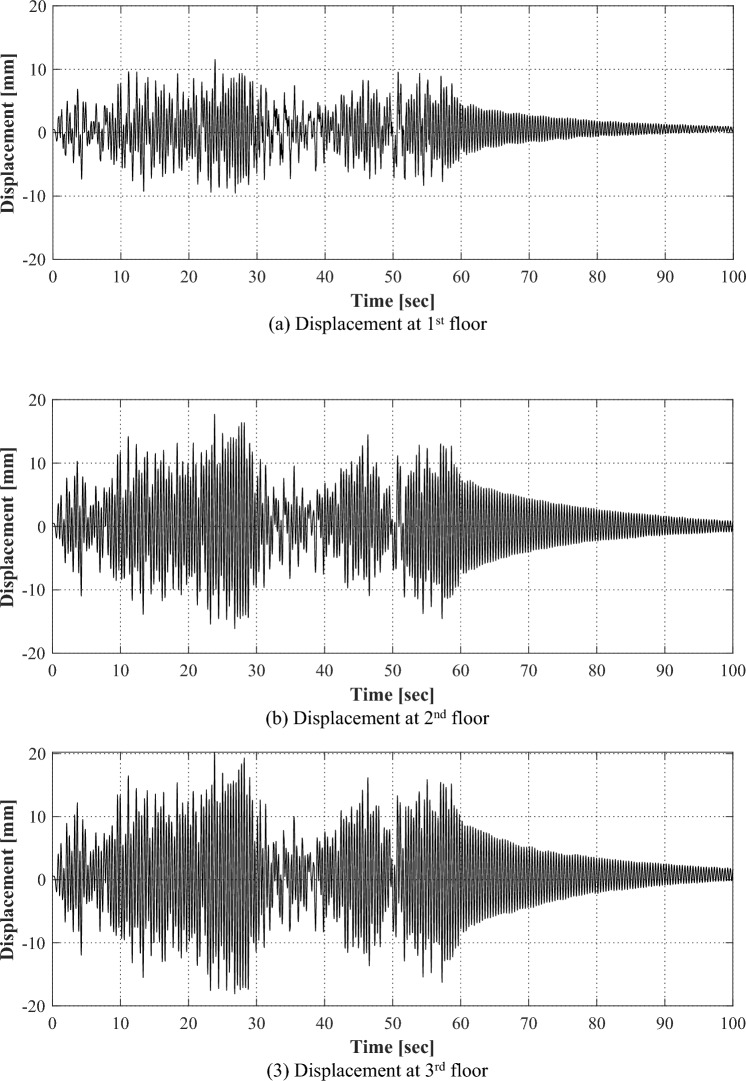
Time series responses for each floor in an intact condition.

Figure 7 shows the displacements of each floor of the structure in intact condition for the applied ground motion. The structure fluctuates for about 60 s where the ground motion is applied, and thereafter, it shows a gradual dissipation of the excitation due to the inherent damping of the structure itself. The maximum displacements in each floor were 11.56 mm, 17.70 mm, and 20.23 mm, respectively. Since the response is caused by the input applied to the structure and the mechanical characteristics of the structure, the structural properties can be identified by analyzing the ambient vibration responses.

**Figure 8:**
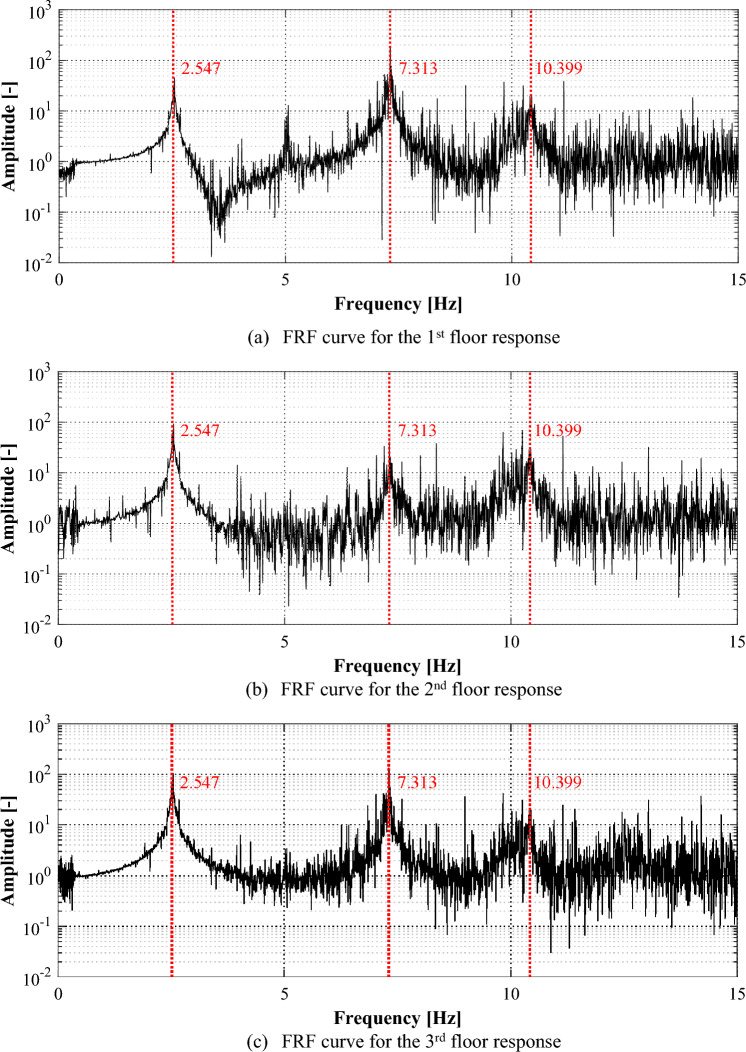
Frequency response functions for the responses in an intact condition.

Among the various methods for analyzing time series data in the frequency domain, this study used the FRF of the responses for identifying the frequency domain characteristics of the structure. Figure 8 shows the FRFs for the responses of each floor. The peak points in FRFs can represent the natural frequency of the structure, and three points can be identified in the figure. The first peak point, that means the first natural frequency, appeared most clearly. Although the second point, which represents the second vibration mode of the structure, was not well-recognized in the FRF of the second-floor response, it could be identified from the other responses. There is a relatively large amount of noise included near the third natural frequency, but it was possible to identify the peak points from all the responses.

**Figure 9:**
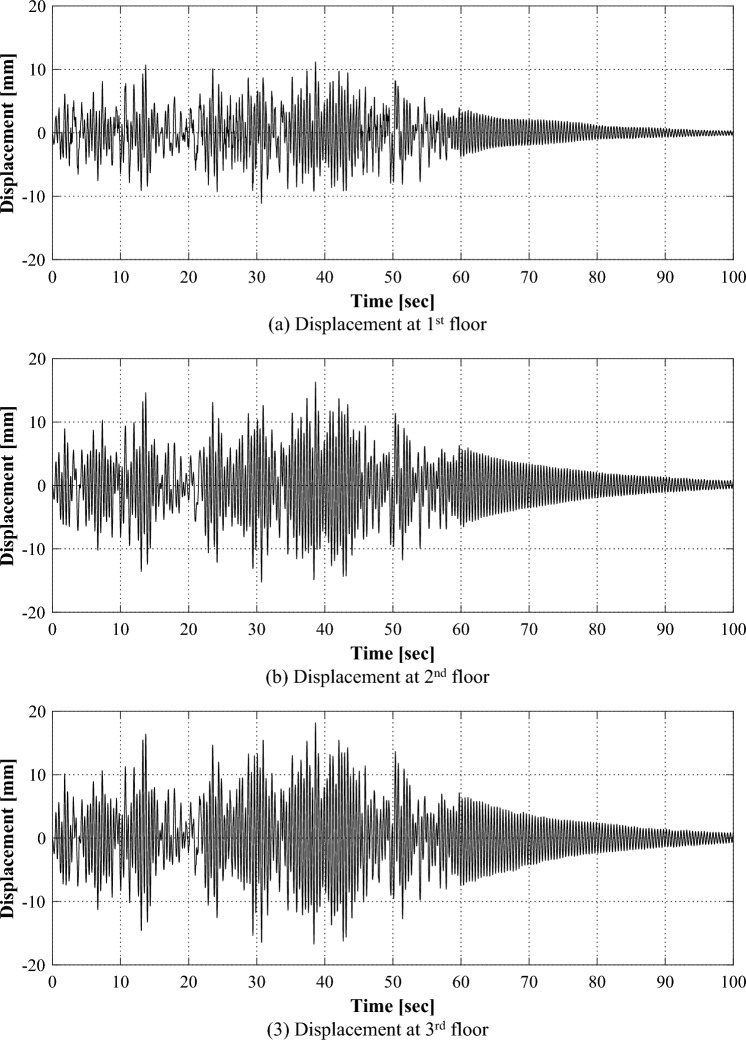
Time series responses for each floor in a damaged condition.

Figure 9 shows the responses of each floor when ground motion is applied to the structure in which the damage is represented by reducing the wall thickness of one side of the first floor. The maximum displacements of each floor appeared 11.18 mm, 16.31 mm, and 18.19 mm, respectively. Compared to the responses of the structure in an intact condition as in Fig. 7, it is considered that the characteristics of the damaged condition are difficult to be identified through the time series responses. Rather, the structural response in the intact condition exhibited a larger magnitude compared to the damaged condition.

**Figure 10:**
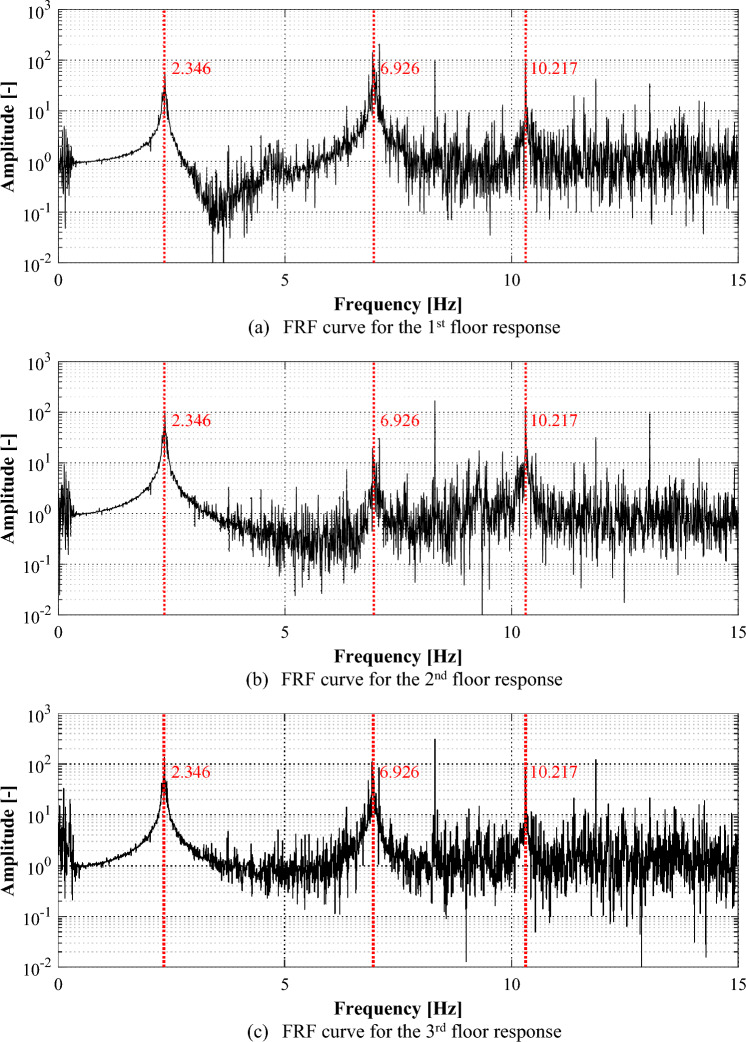
Frequency response functions for the responses in a damaged condition.

Figure 10 shows the FRFs of the responses to analyze the frequency domain characteristics of responses of the structure in the damaged condition. Similar to the results of FRFs for responses of the structure in the intact condition, it was observed that as the frequency range increased, the FRFs contained multiple noise components. However, it appeared that each peak point could be identified from all responses. The natural frequencies of the structure obtained from FRFs for responses of the structure in intact and damaged conditions are summarized in Table 1. The natural frequencies of the structure in the damaged condition appeared with 7.89%, 5.29%, and 1.75% reduced results, respectively, compared to those in the intact condition.

**Table 1:** Natural frequencies of target structure.

	Natural frequency		
	1st mode	2nd mode	3rd mode
Intact condition	2.547 Hz	7.313 Hz	10.399 Hz
Damaged condition	2.346 Hz	6.926 Hz	10.217 Hz
Difference	(− 7.89%)	(− 5.29%)	(− 1.75%)

### Finite element analysis

#### FE model description

**Figure 11:**
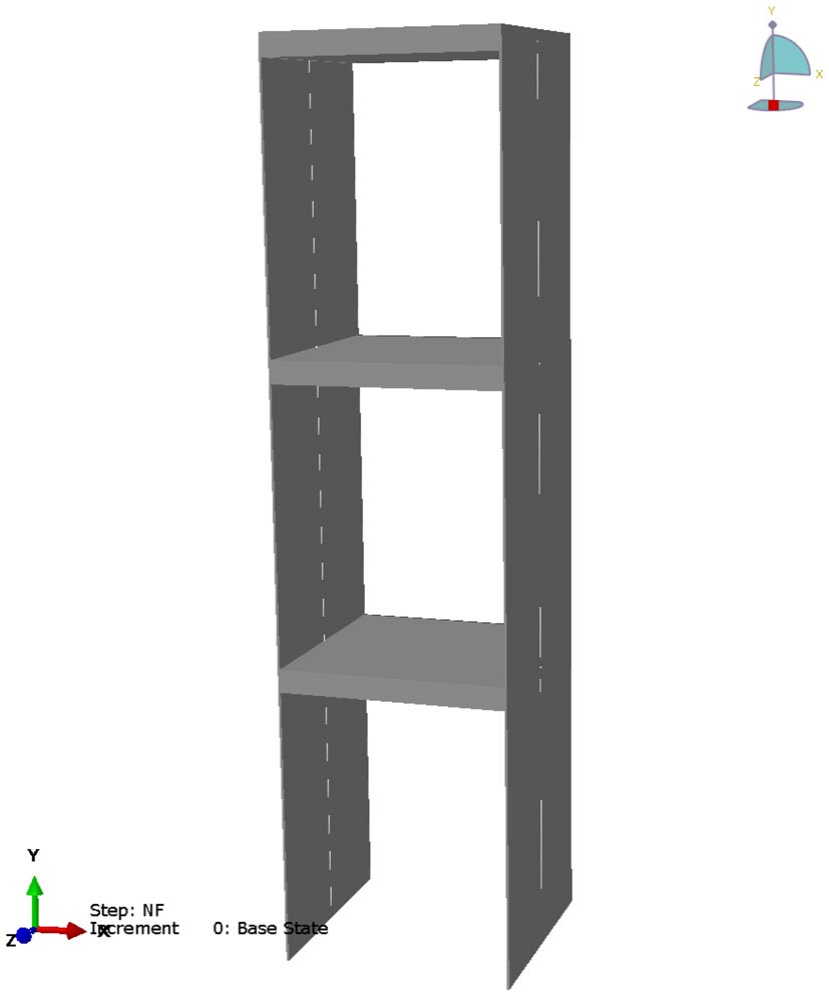
Finite element model for the validation test.

An FE model was constructed for the tested structure to analyze the characteristics of various damaged conditions for the structure. The FE model was modeled with 2D beam elements, since the structure was designed to resist lateral ground motion by the banding behavior of the wall members. FE analysis was conducted using ABAQUS, a structural analysis program, and the constructed model is shown in Fig. 11. The geometrical dimensions of the model are the same as those of the model used in the experimental test, and the values shown in Table 2 were applied to the initial properties for structural analysis.

**Table 2:** Material properties for the initial FE model.

Elastic modulus	Mass density
210 GPa	7850 kg/m^3^

Table 3 shows the mode shapes and natural frequencies of the FE model. The results showed that the cumulative effective mass ratio up to the third mode amounts to approximately 98% for the lateral modes, confirming its sufficiency for analyzing the dynamic characteristics of the structure. However, the FE model cannot accurately replicate the characteristics of the actual structure as it is, since the applied structural properties are different from those in the actual structure. Due to such difference, the FE model is not suitable for analyzing the characteristics of damaged conditions for the target structure, even though it was designed for the structure as a target. The FE model needs to be applied with model updating that optimizes it so that it can represent the characteristics of the target structure.

**Table 3:** Mode shapes and natural frequencies of the FE model with reference properties.

	1st mode	2nd mode	3rd mode
Mode shape			
Natural frequency	2.303 Hz	6.513 Hz	9.512 Hz
Effective mass ratio	(86.42%)	(8.80%)	(2.56%)

#### Reference FE model

To determine the reference FE model that is the basis for analyzing characteristics of damaged conditions for the target structure, a basic optimization process was applied to the initial FE model. The reference model is determined as the FE model that best represents the dynamic characteristics of the target structure through a general model updating process. Note that the reference model is a kind of pseudo model for estimating possible damages through changes in structural dynamic characteristics, and it does not represent the target model itself which can represent other behaviors such as a static behavior of the target structure. The structural dynamic characteristics are dependent on its stiffness and mass. Even for the model to represent a certain dynamic characteristic, there can be numerous possible models with different combinations of stiffness and mass that satisfy it. In order to refer that the reference model is not uniquely determined and can be applied as a variety of models, this study used several reference models for evaluating the applicability of the proposed method.

In this study, the reference models were determined by two procedures. At the first step, the approximate properties of the reference model are determined based on the first natural frequency by regarding the elastic modulus and mass of the entire model as single values. A total of three approximate reference models were determined in this step: the two models were determined by considering one of the initial estimates of the elastic modulus and the mass as constant and adjusting the other variables, and the other was determined as a model having a compromise property between the two models. Table 4 shows the structural properties of the three reference models determined by the first step.

**Table 4:** Structural properties for initial reference models.

Model name	Reference model 1	Reference model 2	Reference model 3
Elastic modulus	210 GPa (constant)	256.88 GPa (derived)	233.44 GPa (derived)
Mass density	6176 kg/m^3^ (derived)	7850 kg/m^3^ (constant)	7013 kg/m^3^ (derived)
1st Natural frequency	2.547		

The second step is the fine-tuning step for the approximately determined reference models. The variables are expanded for each floor, and they are determined to minimize the differences in the 1st to 3rd natural frequencies based on a series of optimization process. The objective function for optimization is as shown in Eq. (6).

6 $$(\{E\}, \{M\})=argmin\left(\frac{1}{m}\sum_{i=1}^{m}{({\omega }_{i}-{\widehat{\omega }}_{i})}^{2}\right)$$

**Figure 12:**
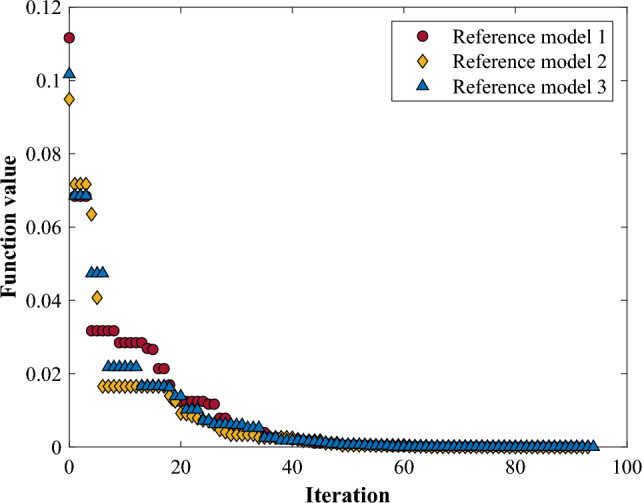
Optimization process for determining reference FE model.

Here, $\{E\}$ and $\{M\}$ are the elastic modulus and mass density of each floor, and ${\omega }_{i}$ and ${\widehat{\omega }}_{i}$ represent the natural frequencies extracted through the experiment of the target structure and based on FE analysis, respectively. $m$ is the order of the natural frequencies to be considered, which was considered up to the 3rd order in this study. The optimization problem at this stage for structural properties with natural frequencies is not much complicated, so there are various methods that can be applied relatively simply. For the fine-tuning of the reference model, the Nelder-Mead method^58^ was applied in this study, and the results of the optimization process for each reference model are shown in Fig. 12.

Although the optimization starts from the initial values that are not different for each floor derived in the previous step, the final values obtained through the process appear differently for the floors. The properties of the reference models derived from the fine-tuning process are shown in Table 5. While the models each have a different distribution of elastic modulus and mass density, they all represent quite identical characteristics in the natural frequencies. Hereafter, the reference model determined in this stage would be then used as a basis that represents an intact condition for training changes in dynamic characteristics caused by damages of the target structure.

**Table 5:** Structural properties for the reference models after fine-tuning.

		Reference model 1	Reference model 2	Reference model 3
Elastic modulus	1F	218.49 GPa	265.65 GPa	238.11 GPa
	2F	198.53 GPa	247.79 GPa	226.30 GPa
	3F	214.14 GPa	263.70 GPa	239.33 GPa
Mass density	1F	6110.04 kg/m^3^	7718.21 kg/m^3^	6957.81 kg/m^3^
	2F	6477.01 kg/m^3^	8284.92 kg/m^3^	7491.52 kg/m^3^
	3F	6033.41 kg/m^3^	7722.37 kg/m^3^	6738.62 kg/m^3^
Natural frequency	1st	2.547		
	2nd	7.313		
	3rd	10.399		

### Damage detection network

This chapter deals with the training of deep learning networks for arbitrarily damaged FE analysis data generated based on the reference FE models determined in the previous chapter. Assuming that there are no changes in mass within the structural damage condition, the training conditions were considered by arbitrarily reducing the stiffness of the wall plate for each floor from 100 to 50%. The training data for the network were generated through FE analysis for models in arbitrarily damaged conditions, compared to the reference models in an intact condition. The number of generated data for the damaged condition is 1000 for each reference model. In the total dataset, 80% was used for training, 10% for validation of the training process, and the remaining 10% for testing.

#### Network configuration

**Figure 13:**
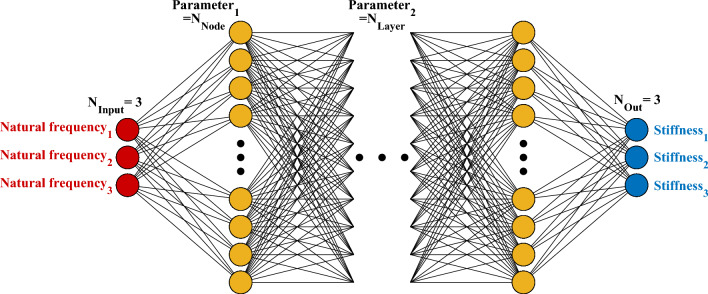
Damage detection network configuration.

A schematic configuration of the training network used in this study is shown in Fig. 13. The input layer of the network consists of natural frequencies from the 1st to the 3rd order, and the stiffness integrities of the wall plates on the 1st to 3rd floors are derived from the output layer. Here, the stiffness integrity represents the ratio of the elastic modulus, expressed as a percentage relative to its distribution in the reference model, with 100% being the maximum value. The relationship between input and output data would be trained by iterative operations passing through hidden layers between the input and output layers. All layers are fully connected to each other to identify the relationships through successive operations. The required number of hidden layers and nodes to serve the network performance depends on the characteristics of the input and output data. In this study, it has been determined through the parametric study in the following section.

#### Parametric study

The training performance of the network depends on various factors. To determine the optimal network suitable for the purpose of the study, this section analyzes the parameters for the hidden layer, which are the significant parameters of the network used in this study. One is the number of hidden layers, which determines the depth and complexity of the network. Increasing the number of hidden layers allows for capturing more intricate relationships in the data, but it also increases the risk of overfitting. Another is the number of nodes within each hidden layer. The number of nodes affects the capacity of the network to learn and represent patterns in the data. More nodes can potentially capture more nuanced features, but an excessive number may lead to overfitting or increased computational complexity. Table 6 shows the values considered in the parametric study. The values were determined as ranges in which the parameters represented sufficient performance and their effects tended to converge.

**Table 6:** Parameters for the hidden layers.

Number of hidden layers	Number of nodes
1	10
2	100
3	250
4	500
5	1000
6	2000
7	3000

In the training process, an epoch represents a single iteration in which the model processes all the dataset once, and there is a requirement to set a constraint on the maximum epoch for proper training termination. Once the maximum epoch is reached, the training process typically stops, regardless of whether the model has fully converged or not. The choice of the maximum epoch value depends on various factors, such as the complexity of the problem, dataset size, and computational resources available. Setting it too low may result in underfitting, where the model fails to capture the underlying patterns in the data. On the other hand, setting it too high can lead to overfitting, where the model becomes too specific to the training data and performs poorly on new data. In this study, the maximum epoch was set to 10,000 by default, which was found to be sufficient for the training performance of the network to converge in the preliminary analysis. Instead, the patience option was applied for an early stop to prevent excessive repetition of the training process. The option refers to waiting for a few more epochs before determining that there is no further improvement in the training process. When the validation accuracy for the validation data applied in the training process does not improve for several epochs, the training would be early-stopped, and the value was set to 200 in this study.

A batch is the number of training data that are processed together in a single forward and backward pass during each iteration. The choice of batch size is a trade-off between computational efficiency and model convergence. A larger batch size can make better use of parallel computing capabilities and speed up training but may require more memory. Conversely, a smaller batch size may provide a more accurate estimate of the gradient but can be computationally slower. In this study, the batch size of 32 was applied, which is a relatively small value, because the problem to be solved through network training is not that complicated.

During the training process, the weights of the model are updated step by step according to the learning rate, which controls the size of the weight adjustment based on the computed gradient. If the learning rate is too high, the model may overshoot the optimal solution and fail to converge. On the other hand, if the learning rate is too low, the training process may be slow, or the model may get stuck in a suboptimal solution. It can also be adjusted during progress using the learning rate schedule option. However, since the dataset and model complexity do not exhibit significant variations in this study, a fixed learning rate of 0.001 was adopted to maintain a stable and consistent update scheme throughout the procedure. It has been widely used as a default value in various deep learning applications, allowing the process to be simplified by eliminating the need for additional hyperparameter tuning and scheduling mechanisms.

When the available data is limited, exhibits imbalance, or shows significant variability that may result in biased training, cross-validation^59^ can be considered to provide a generalized performance evaluation of the model. This study used structural analysis simulation data for training, which were evenly distributed across various conditions. Additionally, the training process was verified by applying a validation dataset that was distinct from the training data, and it was confirmed that overfitting due to bias between the training and validation datasets did not occur. Accordingly, considering that the data was applied relatively clearly to avoid biased network training, cross-validation was not considered in this study.

The reference models have only differences in their material properties, and changes in their dynamic characteristics to the decrease in stiffness appear similar. Therefore, the parametric study was conducted based only on the reference model 1.

**Figure 14:**
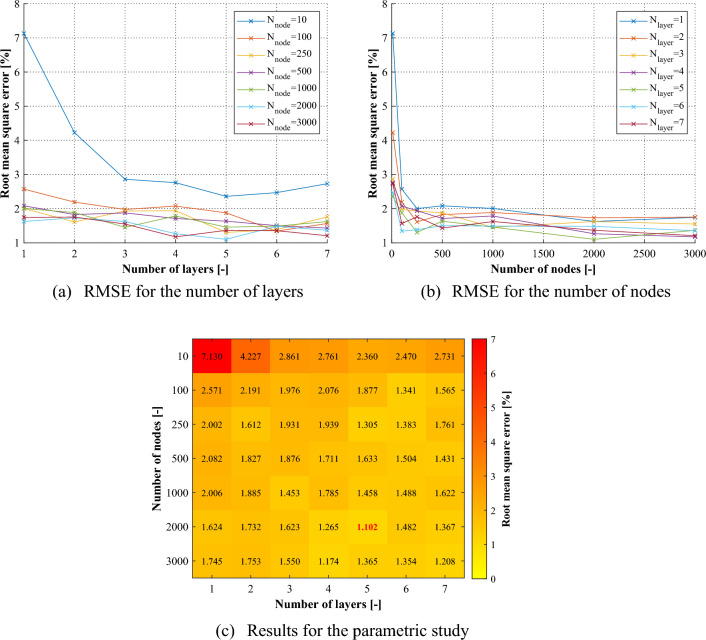
Results for the parametric study on the network configuration.

Figure 14 shows the performance of the network for the considered parameters. As shown in Fig. 14a, the performance of the network tends to converge when the number of hidden layers is about 5, although it varies to some extent depending on the number of nodes in the hidden layer. Figure 14b shows that, while there is a tendency for the network performance to improve to some extent with an increase in the number of nodes, most networks represent satisfactory performance, except for cases where the number of nodes is too small as 10. According to the results of Fig. 14c, this study determined the number of hidden layers and nodes to be 5 and 2000, respectively, for structural damage detection by training the network on arbitrarily damaged FEA data.

#### Training results

The network determined in the previous section was applied to each reference model and trained for the natural frequencies of FE models with the stiffness of each floor arbitrarily reduced from 50 to 100%. The range of stiffness reduction can be adjusted for a comprehensive evaluation of various damage scenarios according to the purpose. A total of 1000 damaged models were generated for each reference model, providing a diverse set of training and testing data. To evaluate the training of the network, 80% of the generated models were used for training the network, while the remaining 20% were reserved for testing its capabilities.

**Figure 15:**
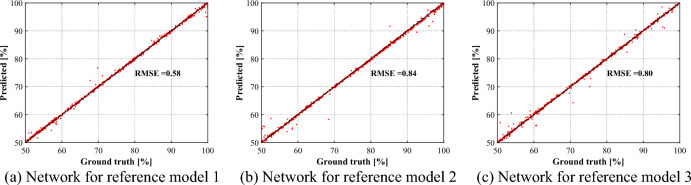
Test results for the trained networks.

Figure 15 shows the results for the tested data. The results demonstrate that the network has been trained adequately, as denoted by the achieved RMSE values of less than 0.85% for all reference models. The low RMSE values indicate a strong correlation between the predicted and actual stiffnesses, further validating that the network can represent generalized performance over a range of stiffness degradations.

**Figure 16:**
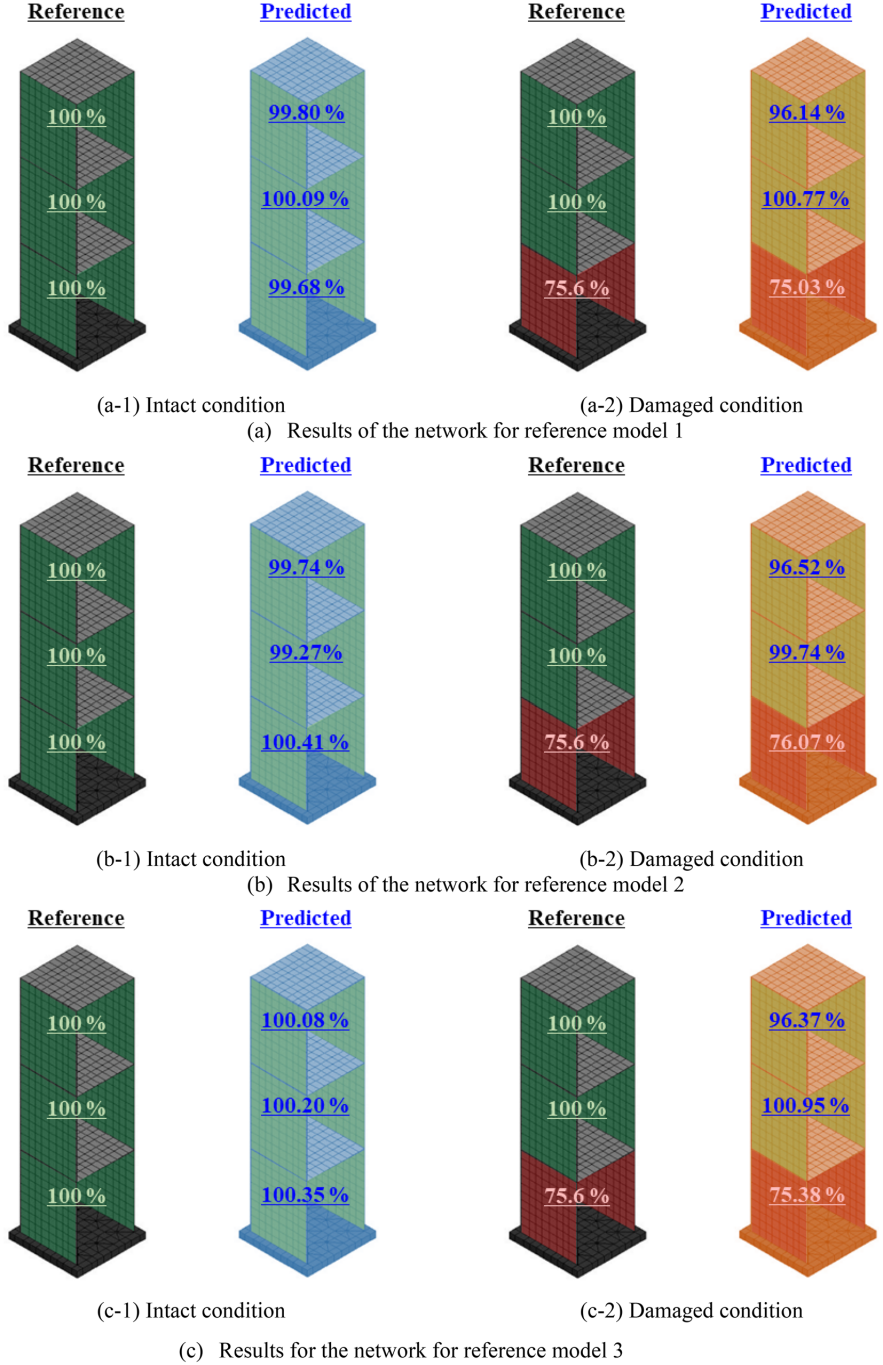
Predicted results for the target structure in the experimental test.

This study adopts the reference FE models to simulate the dynamic characteristics of the structure subjected to damages, as observed in the experimental test. The natural frequencies obtained from the experimental test are applied as inputs for the trained neural networks corresponding to each reference model, and the results are shown in Fig. 16.

The results indicate that all networks yield reasonably accurate estimations of stiffness when the natural frequencies of the structure in an intact condition are applied. Although the network for reference model 2 exhibits the largest error, estimating the stiffness of the second-floor wall plate as 99.27%, it can be regarded as acceptable error for the purpose of the study.

During the experimental test, the damaged condition of the structure was represented by reducing the thickness of one of the first-floor wall plates by 20%. By neglecting the decrease in mass resulting from the reduced thickness of the wall plate, it can be evaluated as equivalent to 75.6% of the stiffness of the first floor compared to the intact condition. The results for the structure in damaged condition demonstrate that it also can exhibit adequate performance in estimating the stiffness distribution of the structure under such damaged conditions. Although the stiffness of the third-floor wall plate was estimated relatively lower than that of the intact condition, it still signifies that the stiffness is sufficiently secured in comparison to the first floor, in which the actual damage condition was applied. Furthermore, the estimated values for the stiffness of the first-floor exhibit relatively accurate results when compared to their ideal values, indicating promising potential for practical application in structural damage detection.

The results also confirmed that the reference models with different material properties can be used equivalently to estimate structural damage based on the dynamic characteristics of a single target structure. Although they cannot reflect the complete behavior of the structure, they can be used for the analysis of dynamic characteristics dominated by the stiffness and mass of the structure. It indicates that there are various FE models that can represent the dynamic characteristics of a single structure, unless it is to analyze the complete behavior of the structure.

## Conclusion

This study proposed a new damage detection methods using model updating, structural analysis simulation, and deep learning. The deep learning network was used for reliable estimation of various damage conditions, and the FE model updating was used for improving the applicability of simulation-based training data to actual target structure. The proposed method was validated through experimental tests on a three-story frame structure, and the following conclusions were drawn.

I. The reference FE model for structural damage detection derived from the FE model updating can have various structural properties as initial values. This implies the presence of multiple FE models that can effectively represent the dynamic characteristics of the target structure, rather than indicating a failure of the model updating process to converge to a single model.
II. The reference FE model is intended to represent changes in the dynamic characteristics resulting from changes in the structural system, rather than replicating all behaviors of the target structure. It has been confirmed that the generated training data based on such reference model can sufficiently represent the changes in dynamic characteristics caused by structural damages in the target structure.
III. The proposed method showed an average error of approximately 0.29% and 1.58% for the stiffness distribution of intact and damaged conditions of the target structure, respectively, providing evidence of the effectiveness of the proposed method.

Since this study was conducted for a relatively simple three-story frame structure and an extensive level of damage was applied, it was possible to sufficiently identify the damage by using only some natural frequencies as input features. In addition to natural frequency, there are various behavioral characteristics such as mode shape and modal energy that can be used as input features. Moreover, beyond the frequency domain methods applied in this study, other advanced techniques like time-domain methods can be used for additional input features. To address more complex target structures or achieve a higher level of precision in damage detection, it may be necessary to consider employing such additional input features or advanced methods.

## Data Availability

The datasets used and/or analysed during the current study available from the corresponding author on reasonable request.
